# A Support Vector Machine Approach for Truncated Fingerprint Image Detection from Sweeping Fingerprint Sensors

**DOI:** 10.3390/s150407807

**Published:** 2015-03-31

**Authors:** Chi-Jim Chen, Tun-Wen Pai, Mox Cheng

**Affiliations:** 1 Department of Computer Science and Engineering, National Taiwan Ocean University, Pei-Ning Road, Keelung 20224, Taiwan; E-Mail: jc60516@gmail.com; 2 Egis Technology Inc., 2F, No. 360, Rueiguang Road, Neihu District, Taipei 11492, Taiwan; E-Mail: mox.cheng@egistec.com

**Keywords:** sweeping fingerprint sensor, truncated fingerprint, support vector machine, biometric recognition

## Abstract

A sweeping fingerprint sensor converts fingerprints on a row by row basis through image reconstruction techniques. However, a built fingerprint image might appear to be truncated and distorted when the finger was swept across a fingerprint sensor at a non-linear speed. If the truncated fingerprint images were enrolled as reference targets and collected by any automated fingerprint identification system (AFIS), successful prediction rates for fingerprint matching applications would be decreased significantly. In this paper, a novel and effective methodology with low time computational complexity was developed for detecting truncated fingerprints in a real time manner. Several filtering rules were implemented to validate existences of truncated fingerprints. In addition, a machine learning method of supported vector machine (SVM), based on the principle of structural risk minimization, was applied to reject pseudo truncated fingerprints containing similar characteristics of truncated ones. The experimental result has shown that an accuracy rate of 90.7% was achieved by successfully identifying truncated fingerprint images from testing images before AFIS enrollment procedures. The proposed effective and efficient methodology can be extensively applied to all existing fingerprint matching systems as a preliminary quality control prior to construction of fingerprint templates.

## 1. Introduction

Fingerprint identification, one of the most popular biometric technologies, has been widely used in law and business applications during the last decade. The technology is rapidly expanding in a number of new application areas, including identification of suspects, credit card verification services, and daily attendance management for employees [1]. The feasibility of these applications mainly based on distinctive and measurable characteristics of biometrics research. According to previous researches, extremely low possibilities were found for different individuals possessing exactly the same fingerprints [2]. Besides, fingerprints retain stable and classifiable characteristics with respect to increasing age. Under such assumptions, fingerprints could offer an infallible means for individual person identification. Examples of large-scale fingerprint systems could be found in the U.S. government including the US-VISIT’s IDENT program [3] and the FBI’s IAFIS services [4]. Recently, applications of fingerprint identification have been also combined with cloud computing technology [5].

Since traditional optical fingerprint sensors have some shortcomings such as large volume, high cost and lack or portability, alternative approaches to sweeping fingerprint sensors designed for portable devices has gradually increased. Successful products are found in mobile phones, tablet PCs and laptop computers due to the merits of smaller size and low cost, and these built-in sweeping sensors are widely used as the basis for personnel identification in various commercial applications. For a sweeping fingerprint sensor, a fingerprint image is reconstructed from dozens of frames which contain certain limited widths [6]. However, when a user sweeps his fingers along the sensor at a nonlinear speed, the captured image might be truncated and degraded during reconstruction the process [7], thus it becomes difficult to establish accurate reference fingerprint templates for future matching applications. This kind of situation occurs frequently, especially with novice users [8], and the worst part is that the erroneous fingerprint images from the initial stage would be stored in the fingerprint enrolment procedures without any warning. Accordingly, the adoption of truncated fingerprint images would decrease the identification accuracy dramatically, and users might lose their confidence in related products and services. It is known that several different approaches for fingerprint matching applications have been proposed, which include correlation-, image-, minutiae- and hybrid wavelet-based methods [9]. Among these algorithms, minutiae and ridge based approaches provide satisfactory performance during matching processes, but unfortunately, no matter which kind of approach the recognition system employs, enrolled truncated fingerprint images may severely affect the matching processes and directly lead to failure.

Truncated fingerprints caused by inappropriate operations during sweeping processes usually share certain unique characteristics. These noisy features within truncated fingerprints reveal less visibility than distorted images caused by other factors, such as scars and permanent marks on fingers. It can also be observed that the insignificant features of truncated fingerprints may easily disappear after performing image reconstruction and enhancement procedures. Nevertheless, existing detection algorithms [10,11] for fingerprint image analysis always focused on checking the adequate feature number of minutiae/ridges [12,13] or image qualities [14,15,16], and most of algorithms spend a lot of time on determining the validity of a fingerprint [17,18,19]. For example, one of the most reliable fingerprint quality inspection systems is the Fingerprint Image Quality (NFIQ) [20] which was developed and maintained by National Institute of Standard and Technology (NIST) in United States. Although NFIQ 2.0 was developed by NIST in 2012, the currently available version is still NFIQ 1.0 and it was applied in this study. To understand the relationship between quality aspects defined by NFIQ and performance of fingerprint recognition, we have collected 43 truncated degraded fingerprint images for system evaluation. Table 1 lists the results of our quality assessment of these truncated fingerprints classified by the NFIQ system. Only one of them was defined within the fifth class, a class of the poorest quality level, and most of the collected degraded fingerprints were classified either in the second or the third classes. According to previous experimental reports, a correct identification rate of AFIS developed by Neurotechnologija Ltd. (Vilnius, Lithuania) was higher than 97.8% for fingerprint images possessing qualities at least classified as the third level, and the detailed recognition rates are shown in Table 2 [21,22]. However, based on the evaluation of 165 experimental tests on our collected truncated fingerprints, if these truncated fingerprint images were enrolled in the AFIS, the successful recognition rate would be decreased to 49% despite the fact most of the fingerprint images were defined with qualities better than the third level. It shows that truncated fingerprint images might be defined as high quality patterns, but these degraded images are extremely harmful if used in practical applications. To solve this problem, we propose a low time complexity and high accuracy identification algorithm for detecting inappropriately scanned fingerprint images, especially for truncated patterns caused by nonlinear finger sweeping speed of users. These types of truncated fingerprints possess clear and specific characteristics such as missing a segment in the middle part of a fingerprint and discontinuous features interspersedly appearing between two truncated subimages. All these features will be discussed, classified, and systematically identified in this paper. Among them, two features were applied for fingerprint quality measurement, including orientation field and ridge thickness [14]. Other features are novel characteristics that are newly proposed in this study.

**Table 1:** Distribution of collected 43 truncated fingerprints according to NFIQ measurement.

Quality Classes	Number of Truncated Fingerprints
1. excellent	0
2. very good	22
3. good	20
4. fair	0
5. poor	1

**Table 2:** Prediction accuracies on (a) left index; (b) right index at various NFIQ levels. (False alarm rate: fingerprints were matched incorrectly; True alarm rate: fingerprints were matched successfully. Data from NIST [22]).

(a) Prediction Rates for Left Index					
Quality	1 (Excellent)	2 (Very good)	3 (Good)	4 (Fair)	5 (Poor)
False alarm rate	0.0136	0.0114	0.0106	0.0076	0.0147
True alarm rate	0.995	0.994	0.978	0.874	0.651
**(b) Prediction Rates for Right Index**					
**Quality**	**1 (Excellent)**	**2 (Very good)**	**3 (Good)**	**4 (Fair)**	**5 (Poor)**
False alarm rate	0.0199	0.0175	0.0176	0.0143	0.0210
True alarm rate	0.994	0.990	0.986	0.924	0.778

To identify true truncated fingerprints and reject misrecognized cases caused by pseudo-truncated fingerprints prior to the AFIS enrollment processes, an automatic and comprehensive detection system for identifying truncated fingerprints is developed in this study. The proposed system analyzes truncated characteristics on each line image and computes its corresponding scores for selected features. If the feature scores satisfy the default threshold settings, the corresponding features would be further sent to an additional classifier based on the supported vector machine (SVM) technique, a widely used machine learning tool, for reconfirmation. As a result, users would be required to perform the fingerprint scanning processes again if the final validation from the proposed SVM classifier confirms the query fingerprint as a true truncated pattern. Machine learning is a subfield of applied statistics, which trains on a collected sample dataset and generalizes rules from previous experiences for later classification applications. The training data with unknown probability distribution is usually applied to extract some general principles and perhaps the distribution for future prediction on new testing data. Several types of machine learning algorithm can be categorized based on trained inputs or desired outcomes, such as supervised, unsupervised, semi-supervised, and reinforcement learning mechanisms. Recently, the SVM kernel method, a supervised learning model, has become one of the most popular classification algorithms for training known features and has been adopted in biomarker identification processes [23]. To construct such a classification model, both positive and negative data classes should be provided as training examples in advance, and a trained SVM model is then constructed according to the selected features. In the feature space, all learning objects are divided by a hyperplane with a separable margin as wide as possible, and the query objects are mapped into the same feature space and assigned to one of the two defined categories based on the locations of the testing objects. In this study, we adopted an SVM classification tool (LIBSVM) developed by Lin’s lab [24,25] to enhance our prediction on query fingerprints. Both normal and truncated fingerprints with defined features were trained and an SVM model was constructed for classifying all new and independent testing fingerprints. The selection of classification features, evaluation on different kernel transformation techniques, and prediction results on benchmark datasets will be shown and discussed in details in the following sections.

## 2. Database and System Model

### 2.1. Database

The experimental fingerprint dataset collected by Egis Technology Inc. (Taipei, Taiwan) contains a total of 2796 fingerprints which were acquired from 19 different users through the EgisTec ES603 sensor. Image resolution is designed as 194 × 4 dots per inch (dpi), which corresponds to the built in sensor of an Acer TravelMate P643 laptop. All fingers swept across the sensor 15 to 16 times, respectively, for each individual. Among them, a total of 43 truncated fingerprint images were be carefully and crossly validated by experts. Table 3 displays the distribution of quality levels computed and defined by the NFIQ software. In the collected 2796 fingerprints, 2774 fingerprints (98%) were classified into either the first (excellent) level or the second (good) level of quality. The results have shown that the collected fingerprint image dataset possesses good quality in general.

The defined 43 truncated fingerprints belong to 12 different fingers from seven users, and these truncated fingerprints could be further categorized into two groups: the first group containing two or more truncated fingerprint images from each finger simultaneously, while the second group containing only one truncated image for each finger. Thus, a total number of 39 truncated fingerprint images belonging to eight different fingers were categorized in the first group and the remaining four truncated fingerprint images from four different fingers were denoted as the second group. Once the query fingerprint with truncated features is firmly identified it will be rejected in the following enrolling registration procedures. On the other hand, if the query fingerprint image has passed all the validation rules in all aspects, the system returns a successful scanning message and the fingerprint image will be enrolled for further recognition applications.

**Table 3:** NFIQ defined qualities for 2796 collected fingerprints.

Quality Levels	Total Count
1. excellent	633
2. very good	1958
3. good	183
4. fair	6
5. poor	16

### 2.2. System Model

**Figure 1:**
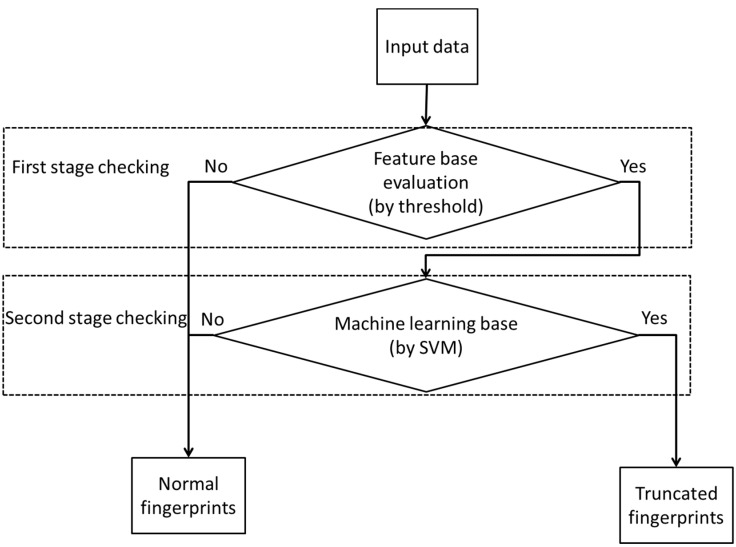
Flowchart of the proposed system for identifying truncated fingerprints.

In this paper, we have designed a system for the automatic detection of truncated fingerprints. Prior to the detection procedures, the system performs a binarization operation on each pixel in the fingerprint based on adaptive thresholding methods. The developed system contains two stages for detecting truncated fingerprints. In the first stage, we employed several checking combinations for enumerating truncated fingerprint candidates. For the second reconfirmation stage, the system cooperated with an SVM tool (LIBSVM) to classify pseudo-truncated fingerprint from previously detected candidates. Once a query fingerprint is firmly identified according to the proposed truncated features at the last stage, it would be rejected immediately before the enrollment registration procedures. On the other hand, if a query fingerprint image has been confirmed as a normal fingerprint either at the first or the second stage, the system returns with a successful scanning message and the reconstructed fingerprint will be formally enrolled for further recognition applications. The flowchart of the proposed system is shown in Figure 1.

## 3. Automatic Detection of Truncated Fingerprints

**Figure 2:**
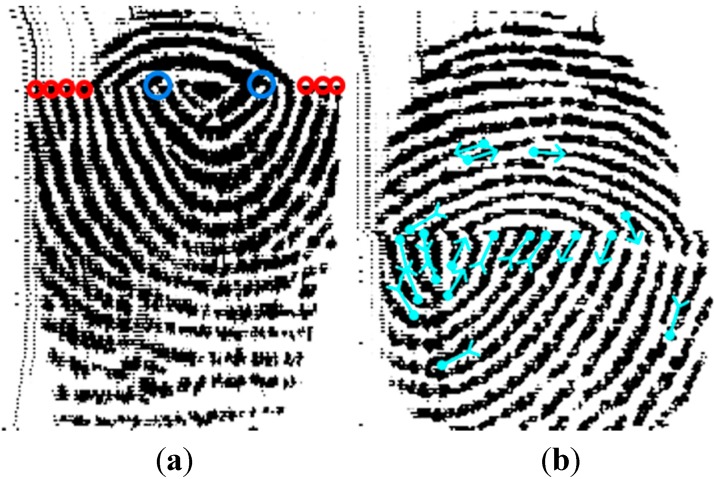
(**a**) Truncated fingerprint contains two types of feature points including pseudo bifurcation nodes (blue circle) and pseudo ridge ending points (red circle); (**b**) Truncated fingerprint possesses minutiae points found by SourceAFIS.

Though a truncated fingerprint appears with discontinuities and/or missing segments in a reconstructed image, it has high probabilities of pseudo feature creation, such as pseudo bifurcation points and pseudo ridge ending features. Figure 2 shows two examples of truncated fingerprints containing pseudo features which were detected as fingerprint minutiae features for identification applications. The left image in Figure 2 is a true truncated fingerprint and its feature points are marked by two colors: blue circles represent examples of pseudo bifurcation nodes; red circles for pseudo ridge ending points. If these two type pseudo features were identified and successfully enrolled as fingerprint minutiae points by a recognition system, it would cause error detection in any subsequent application. To demonstrate how these pseudo features were created, we applied the fingerprint identification software SourceAFIS [26] as a validation tool. It is obvious that the next line image of a missing segment appears with a large number of pseudo minutiae points and it will be enrolled by AFIS. These minutiae points found by SourceAFIS were illustrated in the right fingerprint of Figure 2 with blue marked notations. Unfortunately, the successful recognition rates for fingerprint identification in following applications would drop considerably because of improper enrollment.

A truncated fingerprint with lost segments usually appears with top and bottom lines from missing segments. Thus, two consecutive lines can be distinguished directly from the scores of truncated characteristics. Hence, the proposed system evaluates the query image line-by-line and compares the proposed truncated features between two scanned neighboring line images.

## 4. Feature Analysis of Truncated Fingerprint

There are three important features considered in our first detection module including orientation field, interlaced black-and-white stripe, and mean absolute error between two neighboring segments. These features are described in the following paragraphs.

### 4.1. Orientation Field

Orientation field is defined as the slope of a fingerprint ridge, and it has been used for fingerprint detection and quality evaluation [14]. The changes in orientation field tend to be slow and vary gradually for normal fingerprint cases. However, in truncated fingerprint cases, due to the missing segments, the changes of orientation field become rapid, especially on the discontinuous regions with mismatched blank areas. In practice, when evaluating neighboring segments points possess highly similar orientation fields if they belong to the same ridge. To simplify the calculation requirements, only three continuing rows in both the up and down directions are selected for slope evaluation and a total of eight different direction angles are defined within the range from 0 to π radian degrees. Each π/8 is encoded as an orientation field interval. If different levels of neighboring slope variation are found in more than two orientation field intervals (*i.e*., equal to a total of π/4 radians), this case could be classified as a mismatched orientation field. Hence, the system module would assign the fingerprint line under evaluation with a significant slope change. The formula of slope change at a fingerprint line is defined as in Equation (1):

(1) $$\text{slope change}=\overset{n}{\underset{j=1}{\sum }}\text{changes}\left(j\right)$$

where *n* is the width of the scanned fingerprint image and *j* is the index of the horizontal line. The slope change for the query line is defined according to accumulated changes for each pixel located within the fingerprint line. For each horizontal pixel, we defined changes(*j*) = 1 if point(*i*, *j*) and point(*i* − 1, *j*) possess slope change more than π/4; changes(*j*) = 0 if point(*i*, *j*) and point(*i* − 1, *j*) possess slope change less than π/4. The coordinate (*i*, *j*) represents the discrete pixel locations in a binarized fingerprint image, and “*i*” represents as the index of vertical direction and “*j*” for horizontal direction.

### 4.2. Interlaced Black-and-White Stripes

When a segment within a fingerprint is truncated during the screening processes, it might result in the creation of blank areas located in truncated regions. In such a case, interlaced black-and-white stripes would appear with a certain amount between upper and lower boundaries between that missing segment. If the black-and-white stripes are not continuous between two consecutive rows, the total number of interlaced points will be increased. The number of interlaced points in a row is described as follows:

(2) $$\text{Interlaced point}=\overset{\text{n}}{\underset{j=1}{\sum }}\text{changes}\left(j\right)\;$$

where *n* is the width of the scanned fingerprint image and *j* is the index of the horizontal line. The interlaced points of the query line are obtained by accumulating the changes for each pixel located within the fingerprint line. For each scanned horizontal pixel, we defined changes(*j*) = 1 if point(*i*, *j*) and point(*i* − 1, *j*) possess different intensities; changes(*j*) = 0 if point(*i*, *j*) and point(*i* − 1, *j*) possess identical intensities.

### 4.3. Mean Absolute Errors

Different segments within a fingerprint possess diverse feature distributions. Hence, missing in between segments may result in discontinuous characteristics at truncated boundaries. The variation can be detected by examining the mean absolute error (MAE) feature. This feature is applied to measure differences between two neighboring regions. The parameter of row distance should be determined in advance for calculating the mean absolute error scores. In this study, a default distance of 10 rows was assumed adequate to evaluating the similarity between two neighboring segments according to the image resolution, and the formula is shown in Equation (3).

(3) $$\text{Neighbouring Similarity}=\overset{\text{n}}{\underset{j=1}{\sum }}\text{changes}\left(j\right)$$

where *n* is the width of the scanned fingerprint image and *j* is the index of the horizontal line. The neighboring similarity of the query line is obtained by counting changes for each pixel located within the fingerprint line. For each scanned horizontal pixel, we defined changes(*j*) = 1 if point(*i*, *j*) and point(*i* − 10, *j*) possess different intensities; changes(*j*) = 0 if point(*i*, *j*) and point(*i* − 10, *j*) possess identical intensities.

## 5. Evaluation on Pseudo Truncated Fingerprints

When the previous feature analysis responds simultaneously with high scores in a query fingerprint, a truncated fingerprint is considered to be detected. However, some normal fingerprint images might also possess similar truncated characteristics and easily be misrecognized as truncated fingerprints. These normal fingerprint images possessing truncated features are called pseudo truncated fingerprints. To prevent incorrect classification by the proposed system, an additional pseudo truncated fingerprint filter is designed in this study. Three additional checking conditions and one rejection criterion were proposed for discovering these exceptional cases. If the result confirms that the query image shows a propensity to a pseudo truncated fingerprint through the re-confirmed checking processes, the system would reject the previous verification as a truncated fingerprint. These additional checking conditions on initially assumed truncated fingerprint candidates are described in the following subsections.

### 5.1. Check Thickness of Fingerprint Elements at Truncated Boundaries

**Figure 3:**
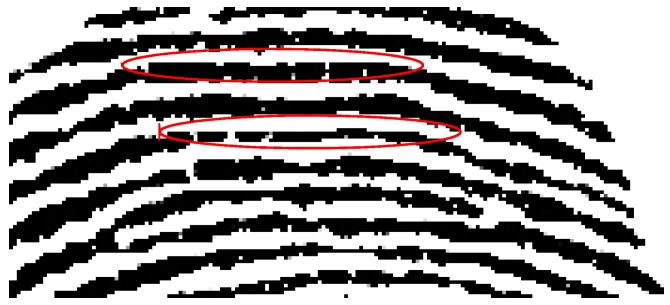
Arch-top patterns of a fingerprint.

Since blank areas could appear on the top of arch patterns, relatively high scores would be obtained regarding the features of interlaced black-and-white changes and mean absolute errors. The detection system might misjudge this type of pattern as truncated regions. An example is shown in Figure 3, and the patterns of arched lines possess similar scores as truncated boundaries. However, these misrecognized boundaries possess another feature that is distinctive from truncated lines, in that the thickness of the detected fingerprint line should be consistently maintained for a detected stroke, and this could be applied to remove misclassified cases from arch-top patterns.

### 5.2. Check Total Number of Continuous Black Pixels

Several cases of arch-top fingerprint lines could not be rejected mainly based on checking thickness of detected elements. This type of fingerprint patterns usually contains long and continuous black pixels with white regions above. Hence, we applied an additional continuous black pixels feature to reconfirm truncated boundary candidates even with a great amount of thickness.

### 5.3. Truncated Fingerprint with Smeared Patterns

**Figure 4:**
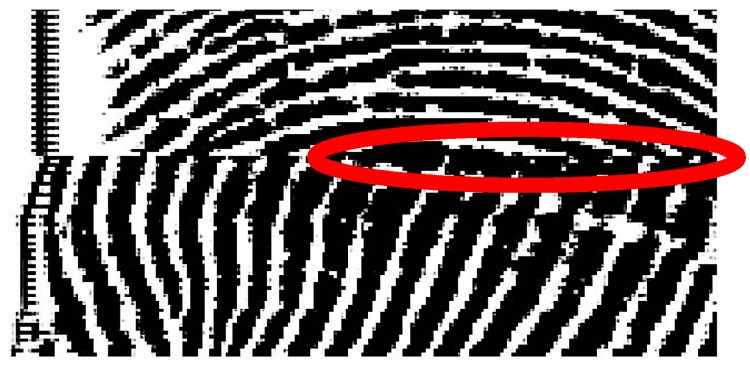
Large segment of continuing black pixels in a row due to smeared noise in a truncated fingerprint.

A truncated fingerprint with smeared patterns usually possesses a great number of continuous black pixels in a row, as the example shown in Figure 4. These kinds of continuous black pixels within a truncated fingerprint might satisfy the previous checking conditions, and it would be rejected as a pseudo truncated fingerprint. Hence, the validation mechanism by inspecting on a total number of continuous black pixels does not apply for an indeed truncated fingerprint with smeared noise. In this designed system, if the total number of consecutive black pixels in a fingerprint line exceeds the default settings, the reconfirmation checking processes will be skipped.

### 5.4. Pseudo Truncated Fingerprints Caused by Scars

**Figure 5:**
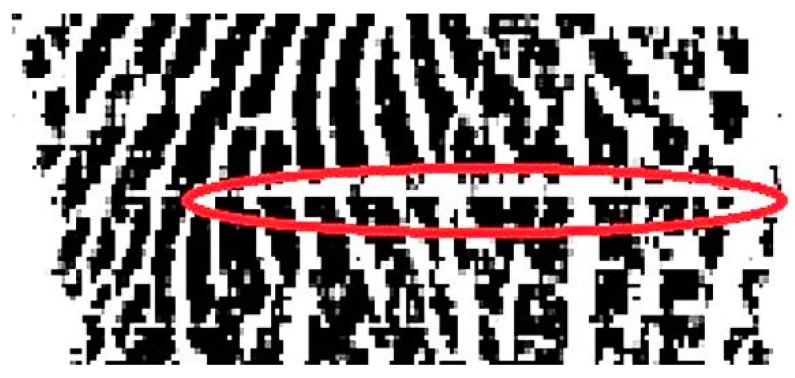
A pseudo truncated fingerprint caused by horizontal scars.

A fingerprint would appear with truncated characteristics when the finger’s surface was scratched and had scars. It is quite often the case that an injured finger appears with truncations like scars on fingerprints. In general, a small scar does not affect the prediction results of the proposed detection system. However, a straight horizontal scar gives high scores when evaluating the proposed truncated features of interlaced black-and-white stripes and mean absolute errors. Patterns of horizontal scars are quite similar to truncated features, and an example of fingerprint with scars is shown in Figure 5.

Intuitively, a scar is relatively easy to be misrecognized as a truncated fingerprint. Nevertheless, by observing carefully, a true scar possesses a feature of alternating pairwise black and white pixels on both sides of the scar, and this important feature could be easily detected by our proposed additional detection module. Hence, pseudo truncated fingerprints could be eliminated from the candidate list and miscalculation rates caused by scar-like patterns can be successfully reduced.

## 6. Experimental Results

In the proposed detection system, there are five sequential validation procedures designed to verify possible truncated fingerprints automatically at the first stage. These modules include: (a) smeared pattern checking; (b) maximum interlaced black-and-white stripe and orientation field change feature checking; (c) orientation field change and ranking of interlaced black-and-white stripe; (d) ranking of oriented field change and interlaced black-and-white stripe feature checking, and (e) comprehensive checking. The default parameters used in this study were obtained by taking the middle values between average values of truncated cases and normal cases. Only the learning dataset was applied for training the default parameter. The order of combinatorial checking procedures is considered based on the time cost of each checking algorithm. Here, we arranged the checking procedures requiring longer time at the later stages. Once the scanned line images in a query fingerprint failed to pass any one of the five validation criteria, the system initiates the next validation stage for the reconfirmation processes. Finally, the system extracts the information of a candidate truncated fingerprint including black-and-white stripe, orientation field change, mean absolute error, thickness of fingerprint element and total number of continuous black pixels as features and passes to LIBSVM for the second stage classification. At the second stage, the system employs the radial basis function kernel type one-class SVM library provided by Lin *et al.* [24]. Once a query fingerprint is firmly identified with truncated features in both the first and the second stages, the proposed system will reject the query fingerprint in the following enrolling registration procedures. On the other hand, if the query fingerprint image could pass all the proposed validation rules at the first stage or be identified as a pseudo truncated fingerprint at the second stage, the system returns a successful scanning message and the fingerprint image would be enrolled in the devices for further recognition applications.

**Figure 6:**
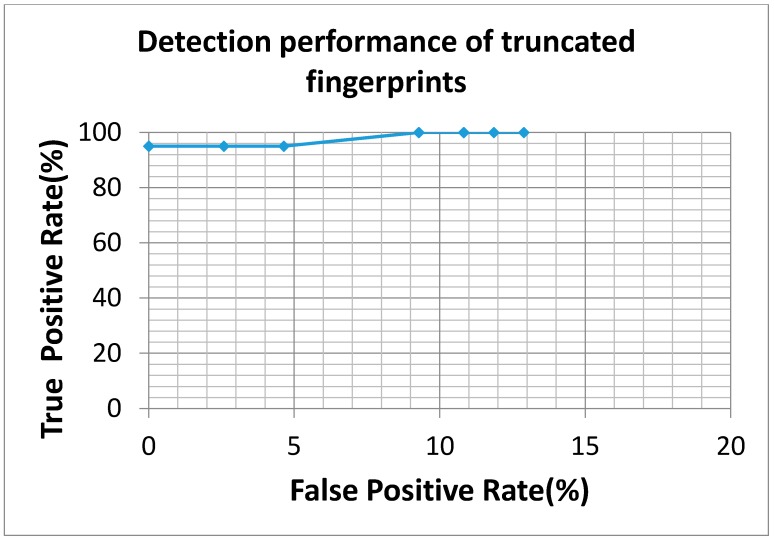
The ROC curve of truncated fingerprint detection is obtained by tuning threshold settings of black-and-white stripe and oriented field change. From left to right, threshold settings were strictly decreased.

**Figure 7:**
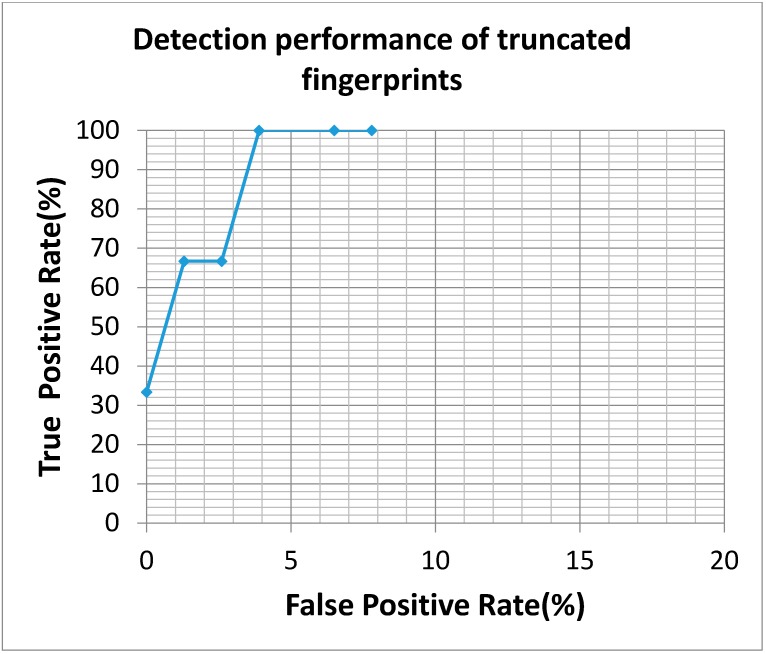
The ROC curve of truncated fingerprint detection is obtained by tuning threshold settings of black-and-white stripe and oriented field change. From left to right, threshold settings were strictly decreased.

This study applied 2753 normal fingerprints in cooperation with 43 truncated fingerprints as a learning dataset for SVM training processes, and another independent dataset collected by Egis Technology Inc. containing 20 truncated fingerprints and 174 normal fingerprints were applied as testing dataset for system evaluation. The SVM training parameters in this study included the cost parameter of 32 and gamma parameter of 0.0064125, respectively. Only the learning dataset was applied for setting these parameters. Each experimental trial required 8.4 milliseconds on average to determine whether the query image is a truncated fingerprint or not, and it showed the advantages of low time complexity in this detection system. Meanwhile, the proposed system achieved an accuracy rate of 99.4% when none of the pseudo truncated fingerprint was classified as truncated fingerprints. Precisely speaking, for all testing fingerprint images, a precision rate of 100%, a recall rate of 95% and a Matthews correlation coefficient of 0.971 were obtained from the proposed system. In addition, as a mean to detect all truncated fingerprints, we have tried to modify threshold settings of black-and-white stripes and oriented field changes. In that case, an accuracy rate of 90.7% could be achieved, and which contained 52.6% precision rate, 100% recall rate, and 0.686 Matthews correlation coefficient. To comprehensively analyze the proposed system, a ROC curve obtained from various experimental settings is shown in Figure 6, and the detecting results compared to NFIQ system are shown in Table 4. In addition, the public database Fingerprint Verification Contest (FVC) 2004 DB3_B [27] has been also verified by our proposed system, and the prediction results are shown in Table 5. The ROC curve obtained from various experimental settings is shown in Figure 7. In the database, only three truncated fingerprints were manually identified and curated, and all truncated fingerprint have been successfully detected and an accuracy rate of 96.2% was achieved.

**Table 4:** The detection results for various systems. We defined that when NFIQ determined a fingerprint belonging to the level 4 (fair) or higher, the system classified the fingerprint as a normal fingerprint. On the other hand, when NFIQ determined the fingerprint at the level 5 (poor), the system classified the fingerprint as a truncated fingerprint.

	NFIQ	Our System	
		100% Precision Rate	52.6% Precision Rate
Detected truncated fingerprint	0	19	20
Undetected truncated fingerprint	20	1	0

**Table 5:** The predicated results for public database FVC2004 DB3.

	Truncated Fingerprint	Non-Truncated Fingerprint
Truncated fingerprint (predicted)	3	3
Non-truncated fingerprint (predicted)	0	74

**Figure 8:**
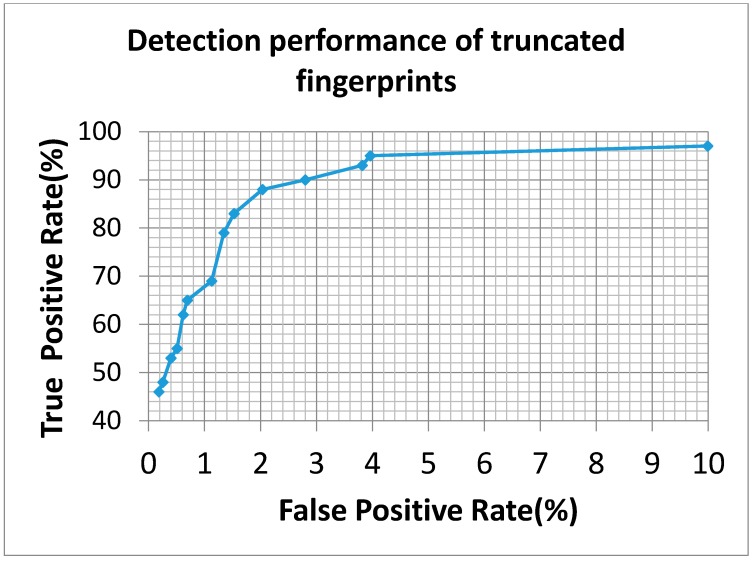
The ROC curve of truncated fingerprint detection is obtained by tuning threshold settings of black-and-white stripe and oriented field change. From left to right, threshold settings were strictly decreased.

We also applied the identical classification approach in the reverse way. We employed the 174 normal fingerprints and 20 truncated fingerprint as a training set and applied the 2753 normal and 43 truncated fingerprints as a testing dataset. The ROC curve obtained from various experimental settings is shown in Figure 8. In this case, the proposed system achieved an accuracy rate of 96% when the recall rate approached to 95%.

## 7. Conclusions and Future Work

An automatic and reliable system for detecting truncated fingerprints is essential for diverse fingerprint identification applications. However, in current marketed products and published papers, there is as yet no comprehensive analytical tool or mature evaluation scheme for detecting truncated fingerprints. The new technology proposed in this paper provides effective and efficient mechanisms to reject truncated fingerprint images before enrolling them into a fingerprint database as verification templates. In particular, the developed system is extremely helpful for inexperienced and beginning users who tend to sweep their fingers on sensors with nonlinear sweeping speeds. It can be noticed that the prevention of enrolling truncated fingerprints in reference databases could increase the accurate recognition rates in all different applications. On average, the proposed system performed comprehensive checking mechanisms on a query fingerprint image within 8.4 ms for each experiment and achieved a successful detection rate of 95% for the testing database, which shows the benefits of the low time requirements and high accuracy of our proposed truncated fingerprint detection system. This system can be directly installed on any embedded device with portable sweeping fingerprint sensors, which only run with limited computer hardware resources, such as small memory, low-power limits, and small device size. The simple and efficient characteristics of the proposed algorithms satisfy the fundamental requirements of portable devices without sacrificing superior recognition performance in all related fingerprint applications.
